# The Impact of Anticoagulation Agent on the Composition and Phenotype of Blood Leukocytes in Dromedary Camels

**DOI:** 10.3390/vetsci9020078

**Published:** 2022-02-13

**Authors:** Jamal Hussen, Turke Shawaf, Sameer M. Alhojaily

**Affiliations:** 1 Department of Microbiology, College of Veterinary Medicine, King Faisal University, Al-Ahsa 31982, Saudi Arabia; 2 Department of Clinical Sciences, College of Veterinary Medicine, King Faisal University, Al-Ahsa 31982, Saudi Arabia; tshawaf@kfu.edu.sa; 3 Department of Biomedical Sciences, College of Veterinary Medicine, King Faisal University, Al-Ahsa 31982, Saudi Arabia; salhojaily@kfu.edu.sa; 4 Agricultural and Veterinary Training and Research Station, King Faisal University, Al-Ahsa 31982, Saudi Arabia

**Keywords:** cell biomarkers, flow cytometry, leukocytes, immunophenotyping, dromedary camel, anticoagulant

## Abstract

For the analysis of several cellular biomarkers, blood samples are anticoagulated using different agents with different modes of action. However, for the most commonly used anticoagulants, EDTA and heparin, varying effects on blood components have been reported in different species. As little is known about the impact of anticoagulants on the immunological evaluation of camel leukocytes, the present study analyzed the leukogram, the immunophenotype, and the cell vitality of camel leukocytes separated from blood samples anticoagulated with EDTA or lithium heparin. Using flow cytometry and staining with monoclonal antibodies to several cell surface markers, the composition and immunophenotype of camel leukocytes separated from blood anticoagulated with EDTA or heparin were analyzed. In comparison to EDTA-anticoagulated blood, using lithium heparin as an anticoagulant resulted in reduced numbers of total leukocytes and reduced numbers of neutrophils, which led to a reduced neutrophil to lymphocyte ratio. The analysis of cell necrosis and apoptosis after the staining of leukocytes with the DNA-sensitive dye propidium iodide and the mitochondrial membrane potential probe JC1 revealed a higher fraction of necrotic neutrophils and higher fractions of apoptotic neutrophils and monocytes in heparin blood than in EDTA blood. In addition, monocytes from heparin blood showed higher expression levels of the cell surface markers CD14, CD163, and MHCII when compared to cells from EDTA blood. Similarly, in heparin blood, CD44 and CD172a were expressed higher on neutrophils, while CD11a was expressed higher on lymphocytes in comparison to cells from EDTA blood. The results of the current study indicate the importance of considering the type of anticoagulant when investigating the composition, vitality, and immunophenotype of camel leukocytes.

## 1. Introduction

The evaluation of immune cell composition and phenotype represents an effective procedure for the identification of disease biomarkers in human and veterinary medicine [1,2,3]. The absolute and relative quantification of the main leukocyte populations including the neutrophilic, eosinophilic, and basophilic granulocytes, lymphocytes, and monocytes, which is called the leukogram, provides a cost-effective evaluation tool with species-specific patterns in health and disease [4]. Furthermore, flow cytometric immunophenotyping of blood immune cells has become an essential tool in current veterinary clinical practice for the diagnosis and prognosis of animal diseases [5].

During recent years, several leukogram changes have been described in healthy and diseased dromedary camel with some discrepancies in the reported leukogram patterns [6]. In these studies, ethylenediaminetetraacetic acid (EDTA) and heparin, which have different modes of action, are the most common anticoagulation agents used for blood sample collection [7]. The metal-chelating agent EDTA acts by chelating free calcium ions (Ca^2+^) in plasma, preventing blood coagulation [8]. On the other hand, the mode of action for the anticoagulation effect of heparin depends on its ability to potentiate the inhibitory activity of the plasma protein antithrombin III, leading to the inhibition of several serine proteases of the coagulation system including factors IIa (thrombin), Xa, and IXa [8,9]. In addition, heparin can act through modulating other protease inhibitors including heparin co-factor II and inhibitors of protein C and plasminogen [9,10].

For both anticoagulants, several effects on cell phenotype and function were described in other species [11,12,13,14]. In a recent report, whole bovine blood samples anticoagulated with heparin showed higher CD14 expression density on monocytes compared to whole blood samples anticoagulated with EDTA [11]. In human neutrophils, heparin caused significant upregulation of CD11b expression with a decrease in CD62L expression compared to EDTA [15,16]. In addition, proapoptotic effect of the anticoagulant on blood leukocytes was described from both heparin and EDTA [8,17]. The molecular mechanisms for the immunomodulatory effects of anticoagulants are not fully understood. Due to the impact of calcium ions (Ca^2+^) on nearly every aspect of cellular life, calcium deficiency has been shown to modulate several processes of leukocyte activation including signal transduction, exocytosis, phagocytosis, motility, and apoptosis [18,19,20,21].

Absolute and differential composition of leukocytes, the neutrophil to lymphocyte ratio as well as the cell-specific phenotype are widely used for the evaluation of animal health status. Therefore, the choice of anticoagulant may have an impact on the immunological evaluation of camel leukocytes and potentially result in interpretation bias. As little is known about the impact of the type of anticoagulant on the composition and phenotype of camel leukocytes, the present study analyzed the leukogram, the immunophenotype, and the cell vitality of camel leukocytes separated from blood samples anticoagulated with EDTA or lithium heparin.

## 2. Materials and Methods

### 2.1. Blood Sampling and Cell Separation

Eight apparently healthy female dromedary camels (*Camelus dromedaries*) aged between eight and 13 years and housed at the research farm of King Faisal University were included for blood collection. Blood samples were collected from each animal by venipuncture of the jugular vein into vacutainer tubes containing EDTA or lithium heparin (BD Biosciences). The samples were transported within one hour from blood collection time to the lab for further analysis. Leukocytes were separated from samples collected in EDTA or lithium heparin tubes by hypotonic lysis of erythrocytes. For this, blood was incubated in distilled water for 20 s followed by the addition of double-concentrated PBS to restore tonicity. This hemolysis procedure was repeated twice until complete erythrolysis. Separated leukocytes were finally suspended in RPMI medium at 6 × 10^6^ cells/mL. The study was approved by the Ethics Committee at King Faisal University, Saudi Arabia (permission number and date: KFU- KFU-REC/2020-09-25).

### 2.2. Analysis of Cell Necrosis and Apoptosis

For the analysis of cell necrosis, leukocytes were labeled with propidium iodide (PI; 2 µg/mL, Calbiochem, Germany), which can only penetrate the cell membrane of necrotic cells and bind their DNA [22]. After flow cytometric analysis using an Accuri C6 flow cytometer (BD Biosciences), PI-positive dead necrotic cells with permeable plasma membranes were distinguished from PI-negative live cells with intact membranes.

Cell apoptosis was analyzed using the mitochondrial membrane potential (MMP) probe JC-1 [23,24]. Leukocytes (6× 10^5^ cell in 100 µL RPMI cell culture medium) were plated in a microtiter plate (96-well). For cell labeling, JC-1 solution (2 μmol/L final concentration) was added to the wells for 15 min at 37 °C. After washing the plates, the cells were suspended in PBS and analyzed using a flow cytometer (BD Accuri C6 flow cytometer). Apoptotic cells (with low MMP and JC-1 monomers) can be identified according to their increased green fluorescence upon excitation with the blue laser at 488 nm. Normal cells, on the other hand, with high MMP and JC-1 aggregates, displayed an orange fluorescence (585 nm, detected in FL-2).

### 2.3. Monoclonal Antibodies

The antibodies used for cell staining are presented in Table 1. All monoclonal antibodies were directed against leukocyte antigens of other animals including lama (CD44 and CD45R), human (CD18), bovine (CD14, CD163, CD4, WC1, CD11a), and swine (MH II). All antibodies were tested for reactivity against camel leukocytes in previous studies [25,26,27,28,29].

### 2.4. Membrane Immunofluorescence

**Figure 1:**
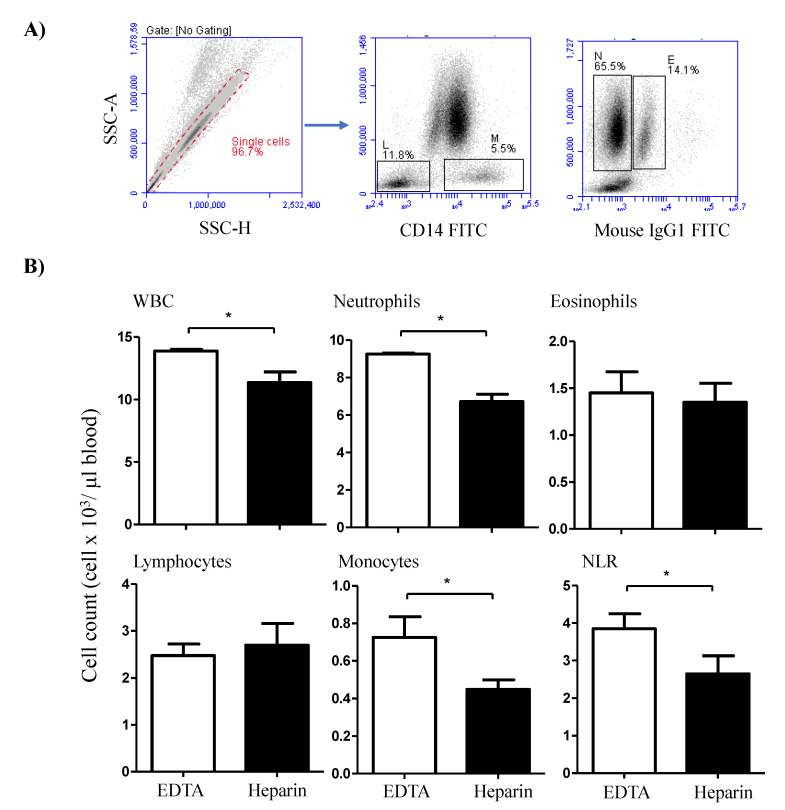
Flow cytometric analysis of the camel leukogram. (**A**) Gating strategy for the identification of camel leukocyte populations. Singlets were excluded from the analysis based on their side scatter height (SSC-H) and SSC-Aria (SSC-A) signals. Within the mononuclear cell population, lymphocytes (L) and monocytes (M) were identified as CD14-negative and CD14-positive cells, respectively. In a SSC-A/ FL-1 dot plot, eosinophils (E) were distinguished from neutrophils (N) based on the higher green autofluorescence of their eosinophilic granules. (**B**) The total leukocyte number was counted under microscope using the Neubauer counting chamber after staining with Türk solution. The relative fractions of leukocyte subsets as determined using flow cytometry were used to calculate the absolute cell numbers. For both heparin and EDTA blood samples, the absolute cell numbers of all leukocyte subsets were presented as the mean and standard error of the mean. Differences between the means were calculated using the t-test and were considered significant (*) if *p* < 0.05.

The immunophenotype of blood leukocytes was investigated using indirect membrane immunofluorescence and flow cytometry [30]. In the first labeling step, 4 × 10^5^ leukocytes were incubated with unlabeled primary monoclonal antibodies (mAbs) specific for the cell surface markers, CD4, WC-1, CD172a, CD14, CD163, MHCII, CD44, CD45, CD11a, and CD18 [6]. After 15 min at 4 °C, the cells were washed twice with MIF buffer and resuspended for the second staining step. After that, the cells were incubated with fluorochrome-labeled secondary antibodies against mouse IgM, IgG1, and IgG2a (Invitrogen). For isotype control staining, class-matching control antibodies (Becton Dickinson Biosciences) were also included. After washing with MIF buffer, labeled cells were analyzed using an Accurie C6 flow cytometer (BD Biosciences) by collecting 100,000 total leukocytes from each sample. Collected data were analyzed with the CFlow Software, Version 1.0.264.21 (BD Biosciences). The total leukocyte number was counted under microscope using the Neubauer counting chamber after staining with Türk solution. The relative fractions of leukocyte subsets as determined by flow cytometry (Figure 1A) were used to calculate their absolute cell numbers.

### 2.5. Statistical Analyses

Statistical analysis was performed using the statistical software Prism (GraphPad). The results were presented as mean ± standard error of the mean (SEM). For each analyzed parameter, the means of the EDTA and heparin samples were compared using the student’s t test and the differences were considered significant if the *p*-value was less than 0.05.

## 3. Results

### 3.1. The Impact of Anticoagulant on the Camel Leukogram

The total white blood cell count (WBC) was significantly higher (*p* < 0.05) in blood samples collected in EDTA tubes (13.8 ± 0.3 cell/µL blood) than in samples collected in lithium heparin tubes (11.4 ± 1.7 cell/µL blood) (Figure 1B). The differential counting of leukocytes revealed higher (*p* < 0.05) percentage and numbers of neutrophils in EDTA (66.5 ± 1.1% of WBC and 9.3 ± 0.3 cell/µL) compared to heparin blood (59.7 ± 2.0% of WBC and 6.7 ± 0.7 cell/µL). The percentage (5.2 ± 0.4% of WBC in EDTA versus 4.0 ± 0.5% of WBC in heparin blood) and numbers (0.7 ± 0.2 cell/µL in EDTA compared to 0.5 ± 0.1 cell/µL in heparin blood) of monocytes were also higher in EDTA blood than in heparin blood. Although the percentages of lymphocytes and eosinophils were higher in heparin than EDTA blood (only significant for lymphocytes), their numbers did not show significant differences between the EDTA and heparin blood samples. The increase in neutrophil numbers with no changes in lymphocyte numbers resulted in a significantly (*p* < 0.05) higher neutrophil to lymphocyte ratio (NLR) in EDTA blood than in heparin blood (Figure 1B).

### 3.2. Lymphocyte Composition Differs between Blood Collected in EDTA and Lithium Heparin Tubes

**Figure 2:**
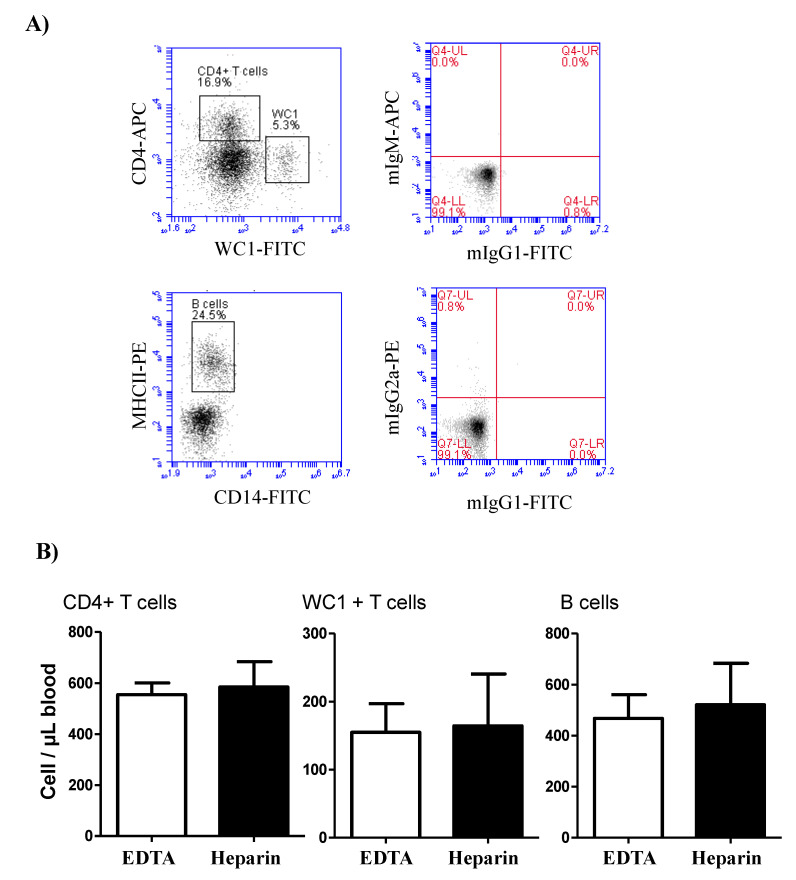
Lymphocyte composition in camel blood collected in EDTA or heparin tubes. (**A**) Gating strategy for the identification of lymphocyte subsets. The whole lymphocyte population was identified within the mononuclear cells in a SSC-A/FSC-A dot plot and the percentage of helper T cells and γδ T cells were identified according to their positive staining with CD4 and WC-1 antibodies, respectively. Camel B cells were identified as MHC-II+CD14-cells in a CD14 against MHC-II dot plot. (**B**) The cell numbers of B cells, helper T cells, and γδ T cells were estimated and presented as mean and standard error of the mean. Differences between the means were calculated using the t-test and were considered significant (*) if *p* < 0.05.

The comparison between blood samples collected in EDTA and lithium heparin tubes revealed no significant (*p* > 0.05) differences in the cell number of the lymphocyte populations, B cells, CD4-positive T cells, and WC-1+ γδ T cells (Figure 2).

### 3.3. The Influence of Anticoagulation Agent on the Expression of Several Myeloid Markers on Camel Monocytes and Neutrophils

**Figure 3:**
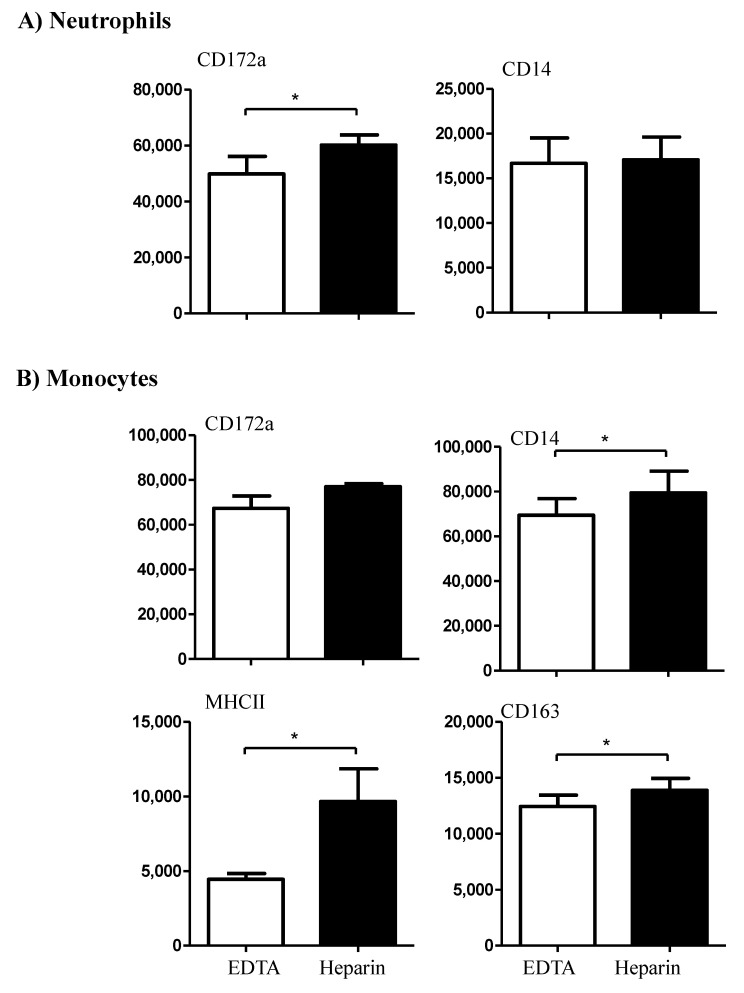
The effect of an anticoagulant on the expression density of some cell surface markers on camel neutrophils (**A**) and monocytes (**B**). Leukocytes were labeled with monoclonal antibodies to CD172a, CD14, MHCII, and CD163, and labeled cells were analyzed by flow cytometry. The expression of each cell marker was evaluated as mean fluorescence intensity (MFI) and the results are presented as mean ± SEM. * indicates a *p* value < 0.05.

While the expression density (mean fluorescence intensity, MFI) of CD14 on neutrophils did not differ significantly (*p* > 0.05) between the cells separated from EDTA or heparin blood, the CD172a molecule was significantly (*p* < 0.05) higher expressed on neutrophils from heparin blood in comparison to EDTA blood (Figure 3A). For blood monocytes, a higher abundance of the cell surface molecules CD14, CD163, and MHCII was observed on cells from heparin blood in comparison to EDTA blood. The expression density of CD172a on monocytes, however, did not differ between EDTA and heparin blood (Figure 3B).

### 3.4. The Influence of Anticoagulation Agent on the Expression Pattern of Several Cell Surface Adhesion Molecules on Camel Leukocytes

**Figure 4:**
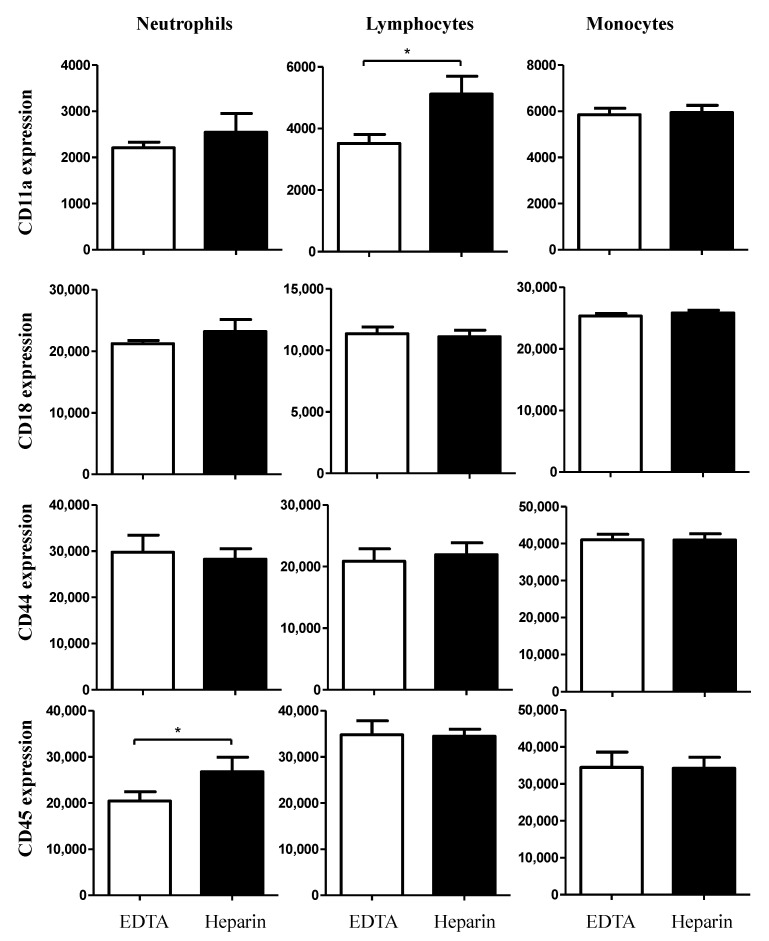
Ex vivo analysis of the impact of an anticoagulant on the expression levels of the cell surface molecules CD11a, CD18, CD44, and CD45 on camel blood neutrophils, lymphocytes, and monocytes. Separated camel leukocytes were labeled with monoclonal antibodies to CD11a, CD18, CD44 and CD45, and labeled cells were analyzed by flow cytometry. After gating on camel neutrophils, lymphocytes, or monocytes, the expression levels of CD11a, CD18, CD44, and CD45 were measured as mean fluorescence intensities (MFI) of the analyzed markers. Data were presented as mean ± SEM. * indicates a significant difference (*p* value < 0.05) between groups, as analyzed by the t-test.

The expression density of the cell adhesion molecule CD11a was only elevated (*p* < 0.05) on lymphocytes from heparin blood compared to EDTA blood, while CD45 was expressed higher on the surface of neutrophils from heparin blood compared to EDTA blood (Figure 4). However, the cell surface molecules, CD18 and CD44 were expressed in comparable levels on the cells from EDTA and heparin blood (Figure 4).

### 3.5. The Influence of Anticoagulation Agent on the Vitality of Camel Leukocytes

**Figure 5:**
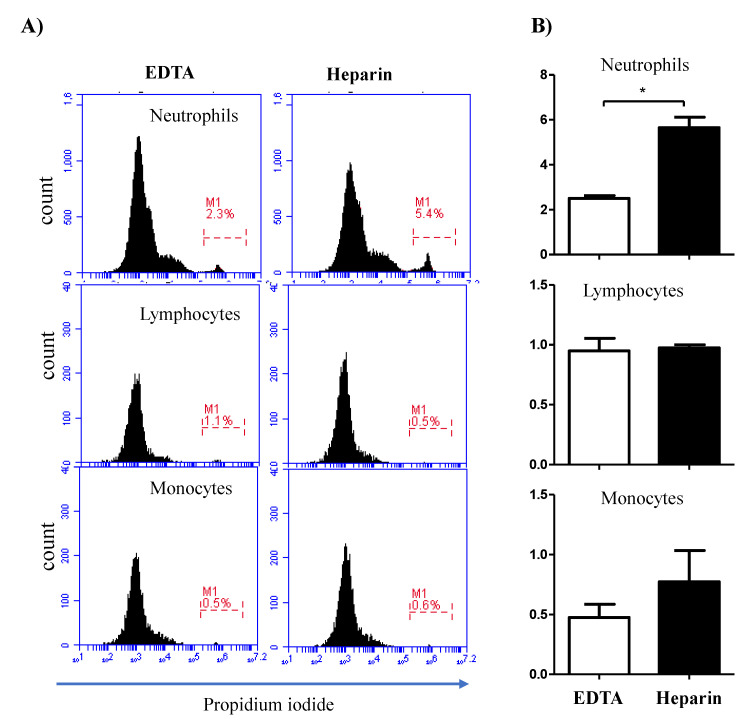
Impact of the anticoagulant on the percentage of necrotic leukocytes. Separated blood leukocytes were labeled with PI and analyzed by flow cytometry. (**A**) Within single cells, granulocytes (G), lymphocytes (L), and monocytes (M) were identified according to their FSC and SSC signals. In a FL3 histogram, PI-negative (viable) cells were distinguished from PI-permeable (necrotic) cells. (**B**) The percentages of necrotic granulocytes, lymphocytes, and monocytes were calculated and presented graphically as mean ± SEM. Differences between the means were calculated using the t-test and were considered significant (*) if *p* < 0.05.

**Figure 6:**
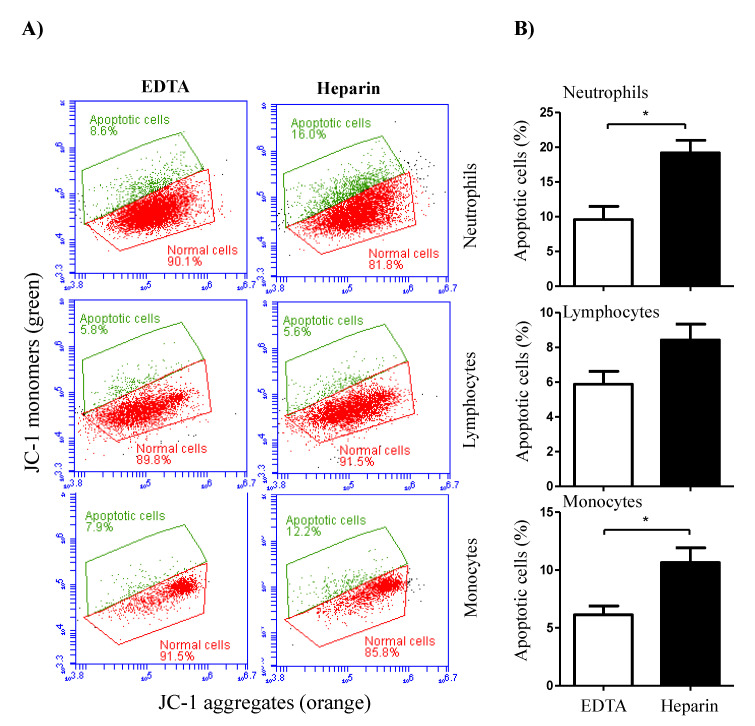
The impact of the anticoagulant on camel leukocyte apoptosis. (**A**) Analysis of cell apoptosis by flow cytometry. After labeling the cells with the mitochondrial membrane potential (MMP) probe JC-1, normal viable cells with orange JC-1 aggregates were detected in FL-2, while apoptotic cells with green JC-1 monomers were detected in FL-1. (**B**) The percentage of apoptotic cells was presented for gated camel granulocytes, lymphocytes, and monocytes as mean ± SEM. (*) if *p* < 0.05.

The analysis of cell necrosis using the DNA-sensitive dye propidium iodide (PI) revealed significant (*p* < 0.05) differences between cell populations in EDTA and heparin blood (Figure 5A,B). The percentage of necrotic (PI-positive) cells within the neutrophil population was significantly higher in heparin blood than in EDTA blood. Although the percentage of necrotic monocytes was slightly (not statistically significant) higher in heparin than in EDTA blood, no difference (*p* > 0.05) in the percentage of necrotic lymphocytes was observed between the two anticoagulants (Figure 5A,B). After excluding the necrotic cells from the analysis, the measurement of cell apoptosis using the mitochondrial membrane potential probe JC-1 revealed a higher fraction of apoptotic neutrophils and monocytes in heparin blood than in EDTA blood (Figure 6A,B). The percentage of apoptotic lymphocytes was, however, only slightly (*p* > 0.05) increased in heparin blood in comparison to EDTA blood.

## 4. Discussion

For several immunological testing procedures including the measurement of leukocyte composition, the immunophenotyping, and the functional analysis of different leukocyte subpopulations, blood samples are collected in tubes containing anticoagulation agents with different mode of actions. However, for the most commonly used anticoagulants, EDTA and heparin, varying effects on blood components have been reported [11,31]. Therefore, the choice of anticoagulant may have an influence on the immunological evaluation of camel leukocytes and potentially result in interpretation bias. As little is known about the impact of the type of anticoagulants on the composition and phenotype of camel leukocytes, the present study analyzed the leukogram, the immunophenotype, and the cell vitality of camel leukocytes separated from blood samples anticoagulated with EDTA or lithium heparin.

In the present study, the lower total white blood cell count (WBC) in heparin blood in comparison to EDTA blood seems to be a result of a decreased cell number of neutrophils. This leukogram pattern resulted in a lower neutrophil to lymphocyte ratio (NLR) in heparin blood than in EDTA blood. The NLR represents an important marker associated with systemic inflammatory responses [32,33,34,35]. As high NLR has been linked to impaired immune cell function [36,37], the lower NLR in heparin blood may lead to false interpretation of the immunological status of the tested camel.

To see whether the different leukogram patterns of EDTA and heparin blood were due to changes in cell vitality, we compared cell necrosis and apoptosis between leukocytes from EDTA and heparin blood. The increased fraction of necrotic neutrophils and apoptotic neutrophils and monocytes in heparin blood in comparison to EDTA blood may have contributed to lower counts of these cells in heparin blood.

The comparable numbers of lymphocyte number as well as the numbers of their subsets in EDTA and heparin blood may be related to the higher resistance of camel lymphocytes to cell stress, which has recently been shown in a heat stress in vitro model [38]. This is also supported by the lack of effect of the two anticoagulants on lymphocyte necrosis or apoptosis.

The analysis of some myeloid markers expression revealed higher levels of CD14, CD163, and MHCII on monocytes separated from heparin blood compared to EDTA blood. As these markers have recently been identified as markers for the characterization of camel monocyte subsets [6,29], the choose of anticoagulant may influence the analysis of camel monocyte composition in health and disease. Due to the role of CD14 together with toll-like receptor 4 (TLR-4) as a bacterial pattern recognition receptor responsible for binding lipopolysaccharide [39], the lower abundance of CD14 on monocytes from EDTA blood may affect the functional responsiveness of camel monocytes to in vitro stimulation with LPS or Gram-negative bacteria.

The surface molecule CD11a, which dimerizes with CD18 to form the adhesion molecule lymphocyte function antigen-1 (LFA-1) [40,41], has recently been identified as a marker of effector lymphocytes in camel [27]. CD44 is a type I transmembrane glycoprotein that is expressed by most cell types including leukocytes and is the major cell surface receptor for hyaluronan (HA) [42,43]. CD172a, which is known as signal-regulatory protein alpha (SIRPa), is a glycosylated cell surface receptor expressed on myeloid cells and functions as a regulatory receptor that inhibits cell signaling [44]. Although the majority of cell surface molecules was not influenced by the type of anticoagulant, the higher abundance of CD172a and CD44 on neutrophils and of CD11a on lymphocytes from heparin blood argues for considering the type of anticoagulant when analyzing the immunophenotype of camel leukocytes.

Although the present study analyzed the impact of anticoagulation agent on the expression of some myeloid and lymphoid cell markers in camels, other important markers could not be included in this work due to the lack of species-specific or cross-reactive monoclonal antibodies. This mainly includes the pan-T cell marker CD3, the cytotoxic T cell marker CD8, the NK cell marker CD335, and the B cell markers CD20, CD21, and CD19.

## 5. Conclusions

The present study identified the significant impact of the anticoagulation agent on the phenotype and vitality of camel blood leukocytes. Using lithium heparin as an anticoagulant for camel blood resulted in higher cell necrosis and apoptosis of neutrophils, reduced numbers of total leukocytes, and reduced neutrophil to lymphocyte ratio (NLR) in comparison to EDTA anticoagulated blood. Monocytes from heparin blood showed higher expression levels of the cell surface markers CD14, CD163, and MHCII when compared to cells from EDTA blood. Similarly, in heparin blood, CD44 and CD172a were expressed higher on neutrophils, while CD11a was expressed higher on lymphocytes in comparison to cells from EDTA blood. Given the importance of the analyzed parameters for the evaluation of the animal health status, the current study indicates the need to consider the type of anticoagulant when analyzing the composition, vitality, and immunophenotype of camel leukocytes and taking those effects in account when interpreting the results. In particular, the reduced NLR in blood samples collected in heparin tubes may be incorrectly linked to good prognosis and disease outcome. In addition, the significant impact of heparin on leukocyte vitality in camel may affect further analysis such as cell separation, cell culture, and functional tests. Therefore, further studies are required to identify alternative anticoagulation agents that can be used with minimal effects on vitality, phenotype, and function of camel leukocytes.

## Figures and Tables

**Table 1:** List of antibodies.

Antigen	Antibody Clone	Labeling	Source	Isotype
CD14	CAM36A	-	Kingfisher	Mouse IgG1
MHCII	TH81A5	-	Kingfisher	Mouse IgG2a
CD172a	DH59b		Kingfisher	Mouse IgG1
CD163	LND68A	-	Kingfisher	Mouse IgG1
CD4	GC50A1	-	Xceltis	Mouse IgM
WC1	BAQ128A	-	Xceltis	Mouse IgG1
CD11a	HUH73A	-	Kingfisher	Mouse IgG1
CD18	6.7	FITC	BD	Mouse IgG2a
CD44	LT41A	-	Kingfisher	Mouse IgG2a
CD45	LT12A	-	Kingfisher	Mouse IgG2a
Mouse IgM	poly	APC	Thermofisher	Goat IgG
Mouse IgG1	poly	FITC	Thermofisher	Goat IgG
Mouse IgG2a	poly	PE	Thermofisher	Goat IgG

MHC: Major histocompatibility complex; WC1: workshopcluster 1; APC: Allophycocyanin; FITC: Fluorescein isothiocyanate; PE: Phycoerythrin; poly: polyclonal.

## Data Availability

The datasets used and/or analyzed during the current study are available from the corresponding author on reasonable request.
